# Contribution of Streptokinase-Domains from Groups G and A (SK2a) Streptococci in Amidolytic/Proteolytic Activities and Fibrin-Dependent Plasminogen Activation: A Domain-Exchange Study

**DOI:** 10.29252/ibj.24.1.15

**Published:** 2019-08-23

**Authors:** Maryam Rafipour, Malihe Keramati, Mohammad Mehdi Aslani, Arash Arashkia, Farzin Roohvand

**Affiliations:** 1 Department of Virology, Pasteur Institute of Iran, Tehran, Iran;; 2 Department of Microbiology, Pasteur Institute of Iran, Tehran, Iran;; 3 Department of Nanobiotechnology, Pasteur Institute of Iran, Tehran, Iran

**Keywords:** Plasminogen, Streptokinase, Thrombolytic therapy

## Abstract

**Background:**

SK, a heterogeneous PA protein from groups A, C, and G streptococci (GAS, GCS, GGS, respectively) contains three structural domains (SKα, SKβ, and SK). Based on the variable region of SKβ, GAS-SK (*ska*) are clustered as SK1 and SK2 (including SK2a/SK2b), which show low and high FG-dependent Plg activation properties, respectively. Despite being co-clustered as SK2a, GCS/GGS-SK (*skcg*) variants display properties similar to SK1. Herein, by SKβ exchange between GGS (G88) and GAS-SK2a (STAB902) variants, the potential roles of SK domains in amidolytic/proteolytic activity and FG-bound-Plg activation are represented.

**Methods:**

Two parental SK_G88_ and SK_STAB902 _genes were cloned into the *Nde*I/*Xho*I site of pET26b expression vector. The two chimeric SKβ-exchanged constructs (SK_C1_: α_G88_-β_STAB_-γ_G88_ and SK_C2_; α_STAB_-β_G88_-γ_STAB_) were constructed by *BstE*II/*BsiW*I digestion/cross-ligation in parental plasmids. SK were expressed in *E. coli* and purified by Ni-NTA chromatography. PA potencies of SK were measured by colorimetric assay.

**Results:**

SDS-PAGE and Western-blot analyses confirmed the proper expression of 47-kDa SK. Analyses indicated that the catalytic efficiency (K_cat_/K_m_) for amidolytic and proteolytic activity were less and moderately dependent on SKβ, respectively. The increase of FG-bound-Plg activation for SK_STAB902_/SK_C1_ containing SK2aβ was around six times, whereas for SK_G88_/SK_C2_ containing skcgβ, it was four times.

**Conclusion:**

Although SKβ has noticeable contribution in FG-bound-Plg activation activity, it had minor contribution in fibrin-independent, amidolytic activity. These data might be of interest for engineering fibrin-specific versions of SK.

## INTRODUCTION

Conversion of inactive Plg into active protease plasmin in blood is the unique mechanism of all PA such as tPA and SK that are used as fibrinolytic drugs for the treatment of life-threatening thrombotic disorders like infarction and stroke^[^^1^^-^^3^^]^. One of the limiting factors of fibrinolytic drugs is their specificity toward fibrin clots, which is important for the bleeding risk of the patients as a side effect of the treatment^[^^4^^]^. In this regard, PA are categorized into two main groups, fibrin-specific and fibrin-independent agents^[^^5^^]^. Fibrin-specific PA, like tPA, target fibrin-bound Plg and act specifically onto thrombus, whereas fibrin-independent ones such as SK have tendency toward circulatory Plg^[^^4^^-^^6^^]^. In fact, SK is not a proteolytic enzyme by itself, and its activity relies on several protein-protein interactions. At first, it binds to Plg and forms a binary 1:1 complex (activator complex) inducing conformational changes in the molecule that results in the generation of an amidolytically activated SK-Plg* complex capable of converting free Plg as the substrate to plasmin (pathway I, conformational activation pathway that is fibrin-independent). Finally, the Plg within the activator complex is converted to plasmin. SK can also bind to plasmin directly with higher affinity, compared with Plg, to form the SK-plasmin complex, which then it converts other Plg substrates to plasmin (pathway II, direct proteolytic activation pathway)^[^^7^^,^^8^^]^*.*

SK is secreted by β-hemolytic Streptococci of the Lancefield groups A (GAS), C (GCS), and G (GGS). SK, as a virulence factor in the streptococcal pathogenesis (especially for GAS) isolated from a moderately virulent GCS (ATCC H46A), was traditionally used as a fibrinolytic drug for decades^[^^9^^,^^10^^]^. SK is a 414-residue protein containing three domains: SKα (aa1-144), SKβ (aa145-287), and SKγ (aa 288-414). SK isolated from different groups of streptococci or even within the isolates of the same groups shows a high degree of heterogeneity at gene and protein levels and results in variations in functional characteristic of SK such as PA potencies and fibrin-specific activity^[^^9^^,^^11^^,^^12^^]^. Identification of SK heterogeneity and its structure-dependent characteristics encouraged the identification of the functional regions in the SK domains for engineering more efficient SK as a thrombolytic drug, especially for enhanced fibrin-dependent activity^[^^10^^,^^13^^,^^14^^]^. SK is the most cost-efficient drug of choice for thrombolytic therapy, particularly in developing countries, and improvement of its therapeutic properties is of high demand^[^^4^^,^^10^^,^^13^^,^^14^^]^. Studies have indicated that the heterogeneity is present in all domains, but the main source of SK variation is mainly in residues 147–218 of β-domain, known as variable one region (SKβ-V1)^[^^11^^]^. According to the phylogenic analysis of nucleotide sequences of SKβ-V1, GAS-*ska* alleles are categorized into two main clusters with different functional features, cluster1 (SK1) and cluster2 (SK2), in which cluster2 is further subdivided into two subclusters, SK2a and SK2b^[^^15^^,^^16^^]^. Of note, SK from GCS/GGS (*skcg*), which exhibit high PA activity in solution (similar to SK1), are categorized into SK2a cluster of the phylogenic tree, indicating the high similarity in the SKβ-V1 of *skcg* and SK2a alleles^[^^15^^,^^16^^]^. Prior studies on functional properties of SK1 and SK2b have demonstrated that SK2b could activate Plg effectively when FG is present, whereas despite exhibiting higher PA potency, SK1 does not require FG for efficient Plg activation^[^^9^^,^^16^^,^^17^^]^. In addition, domain-exchange studies between SK1 and SK2b exhibited the major contribution of SKβ versus minor role of other domains (α/γ) in determining the PA potency^[^^17^^,^^18^^]^. In a recent comparative study, evaluation of the fibrin-dependent activity of recombinant SK form SK2a and SKC-H46A (the commercial source of therapeutic SK) has demonstrated that in the presence of FG, the PA activity of SK2a enhances several folds compared to that of the SKC^[^^19^^]^. However, in none of these prior studies, the fibrin-dependent activity or SK kinetics was/were addressed by domain-exchange strategies, especially in case of SK2a or *skcg *alleles.

We have recently reported the isolation of SK (*skg*) with high PA activity from a GGS (SKG88)^[^^20^^]^. In the present study, using SKG88 (with high PA activity) and a well-known SK2a variants from GAS (SK_STAB902_) with low PA activity^[^^21^^]^, we evaluated the contribution of SK domains in kinetics and FG-bound-Plg activation via “Molecular (SKβ) domain-exchange strategy” between SK genes of these two groups of streptococci. 

## MATERIALS AND METHODS

Bacterial variants

GAS (STAB902) and GGS (G88) with accession numbers CP007041.1 and HM390000.1, respectively were selected as the sources of SK for β-domain exchange. Based on DNA sequences of the variable region of SKβ, SK_STAB902 _and SK_G88_ have been reported as SK2a and SK2a co-clustered-*skcg* alleles, respectively^[^^15^^,^^20^^,^^21^^]^.

Isolation of the SK genes and plasmid construction 

**Fig. 1:**
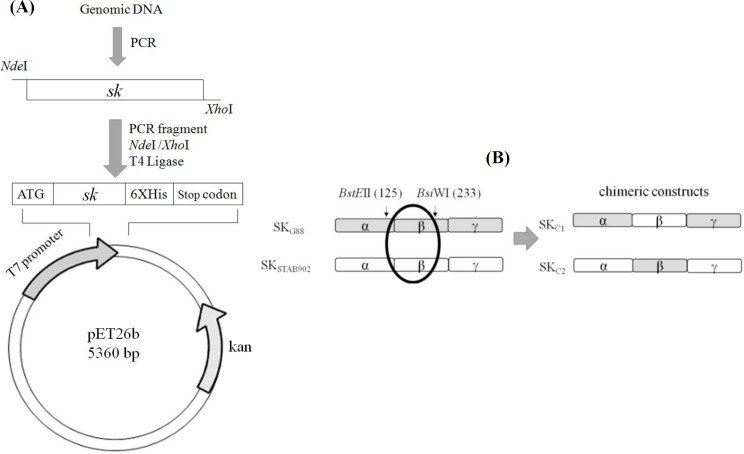
*(A) Schematic illustration for the insertion of *sk* genes into pET26b. Fragments corresponding to *sk* genes were digested with *Nde*I* *and *Xho*I and ligated with the vector pET26b. ATG, start translation codon derived from vector; 6* * His-tag, the tag derived from the vector. (B) Construction of SK*_Cs_* by β-domain exchange (exchange of residues 125 through 233 between parental SK). The unique restriction sites used for sequence exchange are indicated on parental SK.* *SK*_C1_* and SK*_C2_* are made by β-domain exchanges between SK*_G88_* and SK*_STAB902_

 In the first step, to construct the recombinant parent SK, the genomic DNA was isolated by DNA extraction kit (Qiagen, USA). The coding region of *sk* gene (lacking the signal peptide sequence) was amplified by PCR using primers with inserted restriction sites for direct cloning into pET26b vector (forward primer: *Nde*I-SKf: 5׳-GA CGAGACATATG ATTGCTGGACCTGAGTG-3׳; reverse primer: *Xho*I-SKr 5׳-GACACTCGAGTT TGTCGTTAGGGTTATC AG-3׳; the sequences corresponding to restriction sites are underlined). Thermal program was set as 30 cycles of 95 °C for 1 min, 56 °C for 1 min, and 72 °C for 3 min, which was followed by a final extension at 72 °C for 10 min. The resulting amplified fragments were digested with *Nde*I and *Xho*I and cloned into the same sites of pET26b expression vector downstream of T7 promoter and in tandem with the fused C-terminus 6His-tag to yield two parent molecules, pET26b-SK_G88_ and pET26b-SK_STAB902,_ (Fig. 1A). In the second step, to construct the chimeric molecules, the β-domain of parent SK was exchanged. In this context, *SK*_Cs_ (SK_C1_ and SK_C2_) were constructed by *BstE*II*/BsiW*I digestion of the cloned genes in the parental constructs, from nucleotides 375 to 699 of *sk* (the variable region of β-domain was composed of 109 residues) and cross-ligation of the resulting fragments (Fig. 1B). *E. coli* DH5α cells were used for the propagation of plasmids. All cloning steps were performed according to standard procedures^[^^22^^]^. 

Protein expression 

*E. coli *Rosetta strain (Novagen, USA) was used as an expression host for pET26b plasmids according to the manufacturer’s protocol. Briefly, after the transformation of cells with the recombinant plasmids using the standard CaCl_2_ method, expression of protein was induced at OD_600_ of 0.5–0.6 by IPTG to a final concentration of 1 mM. Cells were harvested by centrifugation after three hours of incubation at 37 °C and stored at -20 °C for purification steps^[^^22^^]^.

Protein purification

The expressed SK proteins were purified under native conditions using Ni-NTA affinity chromate-graphy according to manufacturer’s protocol^[^^23^^]^. Briefly, the cell pellets were resuspended in a binding buffer (50 mM of NaH_2_PO_4_, 300 mM of NaCl, and 10 mM of imidazole) with 0.5 mg/ml lysozyme at 2–5 ml per gram wet weight. Following incubation on ice for 30 min, the cells were disrupted by sonication, and supernatant was collected after centrifugation at 10,000 g at 4 °C for 20-30 min. After the addition of 1 ml Ni-NTA resin to the clear lysate, the mixture was shaken at 4 °C for 60 minutes, loaded on column and washed four times with 4 ml of wash buffer (50 mM of NaH_2_PO_4_, 300 mM of NaCl, and 20 mM of imidazole) and four times with 0.5 ml of elution buffer (50 mM of NaH_2_PO_4_, 300 mM of NaCl, and 250 mM of imidazole). 

SDS-PAGE and Western blot analysis 

 The purity of purified SK was analyzed on a 12% (w/v) SDS-PAGE gel, and the concentrations were determined by standard Bradford assay^[^^22^^]^ and OD_280_. For Western blotting, proteins were transferred to nitrocellulose membrane, and the membrane was blocked by 5% BSA. Mouse anti-His monoclonal antibody (Qiagen) was used as the primary antibody, and HRP-labeled goat anti-mouse IgG (Qiagen) as the secondary (tracking) antibody. The bound antibodies were detected using 3,3-diaminobenzidine (Qiagen)^[^^22^^]^.

Determination of SK activity 

 The chromogenic assay is known as an approved internationally standard assay for SK activity (Third International Standard for SK, National Institute of Biological Standards and Controls, NIBSC, 2004, UK). SK activity was determined by using chromogenic substrate, a synthetic tripeptide H-D-valyl-L-leucyl-L-lysine-p-nitroanilide dihydrochloride (S-2251; Sigma, USA)^[^^24^^]^*.* Purified SK proteins (100 nM) were added to a microtiter plate containing 1 mM of S-2251 and 1 µM of Plg (Sigma) at 37 °C in a total volume of 100 µl of the assay buffer (50 mM of Tris-HCl, pH 7.4). Then hydrolysis of S-2251 was measured at 405 nm every 5 min for 60 min in a microplate reader (Synergy 4, UK). To determine fibrin-dependent activity, FG (1 µM) was mixed in a 1:1 stoichiometric ratio with Plg (1 µM) and preincubated at 37 °C for 15 min. SK (100 nM) were added to the mixture, and the change in absorbance at 405 nm was measured at 37 °C after adding S-2251 (final concentration 1 mM). OD at 405 nm was plotted against time and activity rate (slope) was determined from linear portion of the curve. Serial dilutions of Streptase® (CSL, Behring, Germany), a commercially available standard SK, were used to prepare the standard calibration curve based on Hydrolysis of S-2251 by Plg, as described before^[^^24^^]^. All reactions were performed in triplicate^[^^16^^]^*.*

Determination of kinetic constants 

For analyzing amidolytic parameters, stoichiometric concentrations of Plg and SK (5.5 µM SK and 5 µM Plg) were mixed in a 96-well microplate containing the assay buffer (50 mM Tris/HCl, pH 7.5) and incubated at 37 °C for 5 min to construct the SK-PA complex. A suitable aliquot of the complex (final concentration, 100 nM) was transferred to the assay buffer along with various concentrations of S2251 (0.1–1 mM) in a total volume of 100 µl. To determine the kinetic parameters for Plg activation (proteolytic kinetics), 100 nM of SK was added to the assay buffer containing 0.1 mM of S2251 and varying concentrations of Plg (0.3 to 5.0 µM). The reactions were carried out in a microplate reader at 37 °C. The change in absorbance at 405 nm was monitored for 30 min, and the initial reaction rates were obtained from plotting A405/min. The data were plotted as velocity over substrate concentration, and kinetic parameters of Plg activation were determined from Michaelis-Menten (V vs. S) kinetic and inverse (1/V vs. 1/S) Lineweaver–Burk plot using GraphPad Prism 8 (GraphPad Software, San Diego, USA)^[^^20^^]^.

Statistical analyses

Differences of SK activities and kinetic parameters among SK variants were determined using unpaired, two-tailed Student’s* t*-test with 95% confidence intervals. Statistical analysis was carried out using SPSS software version 22.0 (SPSS, Inc., Chicago, IL). All linear regressions were performed applying GraphPad Prism 8, and *p* values less than 0.05 were considered statistically significant.

## RESULTS

Cloning, expression, and purification of the SK

**Fig. 2:**
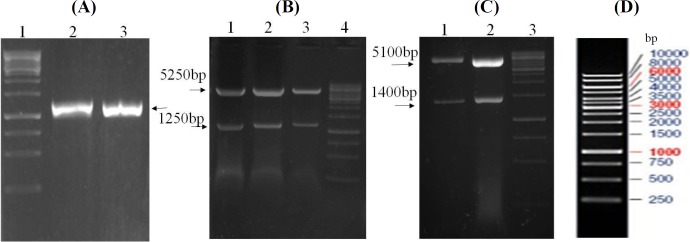
Agarose gel (1%) of amplified *sk* genes and restriction analysis of recombinant pET26b-SK. (A) The coding region of *sk* gene (lacking the signal peptide sequence) was amplified by PCR using primers with inserted restriction sites for direct cloning into pET26b vector. PCR reactions resulted in a single band of the expected length (1250 bp) of *sk* gene. Lane1, DNA marker, Lanes 2 and 3, PCR products of *skg88* and *skstab902 *genes from genomic DNA. (B) Restriction enzyme analysis of the recombinant vector pET26b-SKG_88_, pET26b-SK_STAB902_, and pET26b-SK_C1_ by *Nde*I*-Xho*I yielded 5250 and 1250 bp fragments corresponding to vector and PCR fragments (Lanes 1, 2, and 3, respectively); Lane 4, DNA marker; (C) digested pET26b-SKG_88 _and pET26b-SK_STAB902_ by *Bst*EII produced two bands with the size of 1400 and 5100 bp (Lanes 1 and 2, respectively); lane 3, DNA marker; (D) The size and pattern of DNA markers. The corresponding bands are indicated by arrows, and the sizes of the bands of DNA marker are illustrated on the right. DNA marker (1 kb; Thermo Fisher Scientific SM0311, USA)

**Fig. 3:**
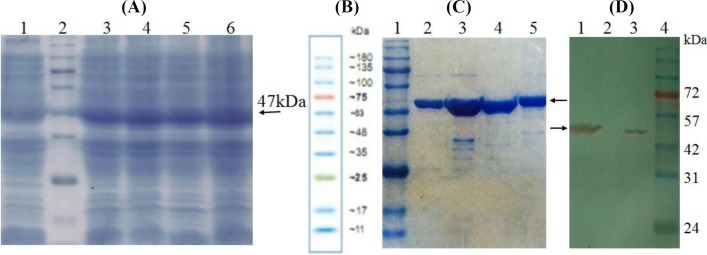
Analysis of expressed proteins by SDS-PAGE and Western blotting. (A) SDS-PAGE (12%) of total protein extracted from *E. coli* Rosetta/pSK_G88_ cells. Lane 1 corresponds to uninduced bacterial cells; lane 2, protein marker 10-180 kDa (SM7012 CinnaGen, Iran); lane 3-6, total protein extracted from IPTG (1 mM) induced *E. coli* Rosetta/pSK_G88_, pSK_STAB902, _and pSK_C1-2_ cells, respectively. (B) The size and pattern of the bands of protein marker 10-180 kDa. *(C) Analysis of purified proteins by SDS-PAGE (12%). Lane 1, molecular weight marker; lanes 2-5, purified SK*_G88_*, SK*_STAB902_*, SK*_C1_*,* *and SK*_C2_* proteins, respectively. *(D) Western-blot analysis of SK_C1_ and SK_C2_ proteins. Lanes 1 and 3, crude lysis of *E. coli* Rosetta cells expressing SK_C1_ and SK_C2_, respectively after induction by IPTG (1 mM); lane 2, crude lysis of *E. coli* Rosetta cells before induction (no band was observed); lane 4, molecular weight marker. The arrows indicate the band of 47 kDa SK

Using the *skf* and *skr* primers and genomic DNA as emplate, PCR reactions resulted in a single band of the expected length (1250 bp) of *sk* gene (Fig. 2A). Cloning steps for the insertion of *sk* gene in pET26b vector is illustrated in Figure 1A. *SK*_Cs_ (SK_C1_ and SK_C2_) were constructed by the exchange of DNA fragments encoding the 125-233 residue fragments between two parent molecules (Fig. 1B). Restriction enzyme analysis of the recombinant vectors harboring *sk* genes (Fig. 2B, 2C, and 2D) and nucleotide sequence analysis (not shown) confirmed the accuracy of cloning procedures. The recombinant parent and chimeric proteins were verified by SDS-PAGE (Fig. 3A and 3B) and Western blotting analyses and the results (Fig. 3D) indicated the presence of the full length protein with expected molecular weight of 47 kDa. Induction of protein expressions in large-scale cultures (50 ml) and purification of His-tagged SK proteins using Ni-NTA affinity chromatography finally provided us with approximately 5 mg of full length proteins with a purity of more than 90% for each protein that was shown by SDS-PAGE (Fig. 3C).

*The steady-state kinetic constants for amidolytic activity*

*To evaluate *the contribution of SKβ on non-proteolytic formation of SK.Plg* complex, the amidolytic activity of SK.Plg* activator complex* was studied. The hydrolysis of substrate S2251 by *SK/Plg* complex* is a measure of non-proteolytic activity.* *The kinetic parameters, including substrate affinity (K*_m_*), *

*catalytic activity (K*_cat_ *), and the constant of catalytic efficiency (K*_cat_ */K*_m_ *;* efficiency of the Plg conversion into plasmin*) were measured (*Table 1*). As shown in *Table 1*, β-domain exchange did not influence the catalytic efficiencies of resultant chimeras significantly; K*_m_* of *SK_C1 _(G88-STAB-G88) *decreased only 10% (from 0.41 mM of parent SK*_G88_* to 0.36 mM); furthermore, the K*_cat_* attenuated 10% compared to that of the parent SK*_G88 _*(from 83.33 min*^-1^* of parent SK*_G88_* to 74.93 min*^-1^*), which resulted in almost equal catalytic efficiency (204.99 vs. 205.84 min*^-1^*/mM). Likewise, while the K*_m_* of SK*_C2 _(STAB-G88-STAB) increased 9% (0.39 vs. 0.36 mM)* and the K*_cat _*enhanced 20%* *relative to the parent molecule, SK*_STAB902 _(from 30.93 min^-1 ^to 36.96 min^-1^) resulted in only 10% raise of catalytic efficiency (94.58 vs. 86.09 *min*^-1^*/mM*; p* < 0.05; *Table 1*.*

**Table 1:** The amidolytic and proteolytic kinetic parameters of SK variants

**SK variants**	**Amidolytic constants** ^a^			**Proteolytic constants** ^b^		
	**Km** **(mM)**	**K**_cat_ **(min**^-1^**)**	**K**_cat _ **/K**_m_ **(min** ^-1^ **/mM)**	**K**_m_ **(µM)**	**K**_cat_ **(min**^-1^**)**	**K**_cat _ **/K**_m_ **(min** ^-1^ **/µM)**
SK_G88_	0.41 ± 0.006	83.33 ± 2.82	204.99	0.77 ± 0.004	19.82 ± 4.20	25.67
SK_STAB902_	0.36 ± 0.007	30.93 ± 0.78	86.09	4.15 ± 0.72	10.64 ± 1.25	2.56
SK_C1_	0.36 ± 0.026	74.93 ± 3.58	205.84	1.09 ± 0.13	22.59 ± 5.02	20.70
SK_C2_	0.39 ± 0.063	36.96 ± 3.75	94.58	3.73 ± 1.23	11.68 ± 4.68	3.12

^a^The amidolytic kinetic parameters of SK variants were determined by pre-complexing equimolar ratios of SK and Plg. ^b^For measurements of proteolytic activities, stoichiometric activator complexes of SK.Plg* were formed by mixing Plg and SK variants. Kinetic parameters including substrate affinity (K_m_), catalytic activity (K_cat_), and the constant of catalytic efficiency (K_cat_/K_m_) of Plg activation were calculated from Michaelis-Menten (V vs. S) kinetic and inverse (1/V vs. 1/S) Lineweaver-Burk plots using GraphPad Prism 8 software. All measured *p *values were less than 0.05 and considered significant. The Table indicates the minimal alteration of amidolytic kinetic parameters, while the proteolytic constants changed more significantly, especially in case of K_m_.

*The steady-state kinetic constants for proteolytic activity*

 The proteolytic activity of SK variants after equimolar SK.Plg complex formation was assayed against a concentration range of substrate Plg*. As shown in *Table 1*, the K*_m _*of SK*_C1 _*raised 1.5fold (1.09 vs. 0.77 µM), and the K*_cat_* raised 13% (22.59 vs. 19.82 min*^-1^*) compared with that of SK*_G88_* that led to 20% lower catalytic efficiency (from 25.67 of parent SK*_G88 _*decreased to 20.70 min*^-1^*/µM). The proteolytic constants of SK*_C2_* were also altered relative to the parent SK*_STAB902. _*The catalytic efficiency of SK*_C2_* increased 20% (3.12 vs. 2.56 min*^-1^*/µM), since the K*_m_* decreased 10% (3.73 vs. 4.15 µM) and the K*_cat _*raised 10% (11.68 vs. 10.64 min*^-1^*) compared with those of SK*_STAB902_* (*p* < 0.05; *Table 1*). *These alterations were in accordance with the change of specific activities and imply that β-domain exchange influences the conformational and functional changes of SK yielding alteration of the kinetic constants of proteolytic pathway and conversion of Plg substrate to plasmin, which led to different activities of SK variants. 

SK activity in absence/presence of FG

**Fig. 4:**
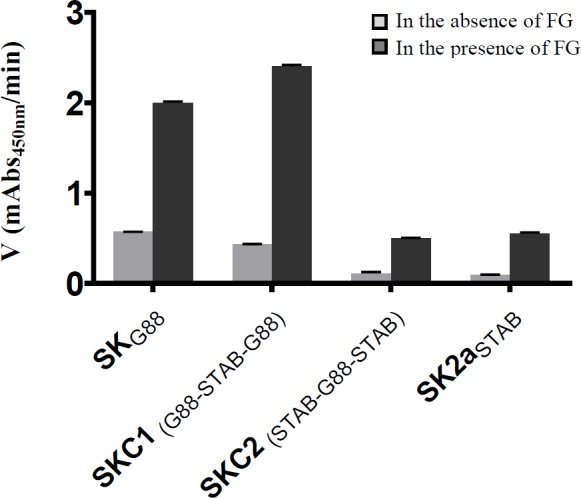
*The* Plg activation rates* of various SK in the presence and absence of FG. The activation rates were measured by monitoring the absorbance at 450 nm and* calculated by linear regression from the linear regions of plots A405 nm vs. time (t). The activation rates of all constructs improved several orders of magnitude. Notably, FG stimulated significantly *(*p* < 0.05)* the activity of SK2aβ containing *SK*_STAB902_* and SK*_C1_ more efficiently than that of *skcg*. *SK*_G88 _*and* *SK*_STAB902 _*are the parental and SK*_C1 _*and SK*_C2_* are the chimeric constructs produced by β-domain exchange*

Employing chromogenic substrate S-2251, the change in absorbance at 405 nm was measured as a function of time. As shown in Figure 4 and Table 2, the activation rate of all constructs raised significantly in the presence of FG, but with different order of magnitudes. The activation rates of constructs owning SK2aβ, namely SK_STAB902_ and SK_C1_, showed 6.1 and 5.7fold increase (0.55 and 2.43 vs. 0.09 and 0.43), whereas the activation rates of the constructs owning *skcg*β, namely SK_G88_ and SK_C2_, enhanced 3.5 and 4.5fold (2.0 and 0.5 vs. 0.57 and 0.11), respectively in the presence of 1 µM FG. It is worth mentioning that as expected, the stimulatory effect of FG on SK2a was greater than *skcg* allele, and the activity of SK_C1_ in the presence of FG was more than that of SK_G88_ *(*p* < 0.05)*.

## DISCUSSION

The rationale for performing the present study was to gain insights into the degree of fibrin dependency and kinetic differences of SK_G88_ from group G streptococci (*skcg* allele) compared with the SK_STAB902 _from cluster 2a, group A streptococci, and the role of β-domain in these characteristics. To the best of our knowledge, there is no prior study on the role of β-domain in fibrin-dependent mode of action or SK kinetics by domain-exchange strategies, especially for SK2a or *skcg *alleles. Our results confirmed the higher increase in SK activities of SK_STAB902_ than *skcg* allele in the presence of FG and indicated the major contribution of β-domain in conferring this feature, which might lead to designing fibrin-specific generations of SK. As shown in Figures 2A and 3, results of PCR and SDS-PAGE/Western blot analyses indicated the expected length (1250 bp) of *sk* gene and SK protein (47 kDa), which is in agreement with prior molecular isolation and expression studies for SK (reviewed in^[^^10^^]^).

**Table 2:** PA activity in the absence and presence of FG

**The parent/** ***SK***_Cs_	**The PA activity in the** ** absence of FG (ΔOD**_405_ **/t)**	**The PA activity in the ** **presence of FG (ΔOD**_405_ **/t)**	**Fold increase of activity in the presence of FG**
SK_G88_	0.57 ± 0.002	2.0 ± 0.015	3.5
SK_STAB902_	0.09 ± 0.006	0.55 ± 0.034	6.1
SK_C1_	0. 43 ± 0. 006	2.43 ± 0.02	5.7
SK_C2_	0.11± 0.001	0.5 ± 0.003	4.5

According to kinetic results (Table 1), the β-domain exchange did not cover any significant alteration in the amidolytic catalytic efficiency (K_cat_/K_m_) of SK.Plg* activator complexes of the *SK*_Cs_ (SK_C1_ and SK_C2_) compared to their parental SK. Indeed, these results revealed a major role for α/γ-domains in determination of amidolytic activity, which is consistent with several prior reports on the potential role of α/γ-domains in interaction with Plg and formation of the SK.Plg* activator complexes^[^^25^^-^^29^^]^. In this context, the critical role of Ile1 in α-domain of SK for the formation of SK.Plg* activator complex through establishing a salt bridge with Asp741 of Plg, which is essential for the induction of an active site in Plg is emphasized^[^^27^^]^. In addition, a surface-exposed loop in residues 88-97 of α-domain has been also reported. This loop not only is involved in SK.Plg* activator complex formation but also interacts with the catalytic domain of Plg (microplasmin). Thus, this behavior might have a crucial role in catalytic turnover of the substrate Plg while minimally affecting enzyme-substrate affinity^[^^25^^,^^26^^]^*. *Accordingly, the potential key role of Arg324, Asp325, Lys332, and Lys334 as well as residues between 314-342 in the γ-domain for the amidolytic activity of the SK.Plg* activator complex has been proposed^[^^28^^,^^29^^]^. 

The K_cat/_K_m_ values of proteolytic activity (as an indicator for the efficiency of conversion of substrate Plg into plasmin) for SK_C1_ and SK_C2_ compared to parental SK showed more significant alterations compared to the amidolytic efficiency (Table 1). Of note, the K_m_ values, which show the substrate affinity, were affected more significantly compared to the K_cat_, indicating the catalytic turnover (which implies the important role of the exchanged segment in affinity of the activator complex to substrate Plg). Our finding is in accordance with several studies addressing the importance of SKβ in the proteolytic activity of SK. Indeed, by bridging the SKα and SKγ, SKβ mediates high affinity interaction between SK and Plg^[^^20^^,^^30^^-^^32^^]^. This domain seems to involve in high-affinity interactions between SK and Plg substrate, as well as strong binding of Plg substrate to the proteolytic complex and efficient conversion of Plg substrate to plasmin^[^^31^^]^. Our results are in line with reports on the critical role of loop 170 of SKβ in mediating catalytic turnover of the substrate Plg (indicated by K_cat_ of the proteolytic pathway)^[^^8^^,^^25^^]^. In further support of our results, the role of loop 250 in SKβ for Plg recognition by active SK.Plg* complex and Plg docking has also been suggested^[^^32^^]^. It should be noted, however, that several residues in other domains of SK, like residues 314–347 and 285–414 of SKγ, have been proposed for contribution in the processing of Plg by the SK- plasmin complex and catalytic activation of Plg^[^^28^^]^.

In general, the presence of FG enhanced the activity of all SK, but its effect was around two times higher for SK2aβ containing SK_STAB902_/SK_C1_ than skcgβ containing SK_G88_/SK_C2_ proteins (6.1/5.7 vs. 3.5/4.5fold enhancement of activity, respectively; Fig. 4 and Table 2). These results accord with prior reports on generally positive effect of the binding of FG to Plg to change its conformation in favor of enhanced activation of Plg^[^^16^^,^^19^^,^^33^^]^, which has been higher for GAS-SK2a variants compared to *skc*-SK from GCS^[^^19^^]^. In further agreement, it has also been reported that the PA activity of SK1 and SK2b variants enhances 1.2-1.8 and 10-18fold, respectively in the presence of FG^[^^33^^]^. However, in these prior studies, the role of SK domains or special residues on this property was not elucidated. Identification of the SK domains involved in FG-bound-Plg activation might help to improve the fibrin-specific characteristics of SK for therapeutic purposes^[^^13^^]^. Consistent with a recent study, SK_G88_, as represented in Figure 4, showed high intrinsic FG-bound-Plg activation^[^^20^^]^, which was about fourfold higher than SK_STAB902_. Interestingly, this high FG-bound-Plg activation further enhanced in SK_C1_, while in the absence of FG, PA potency of SK_C1 _was still lower than the parental SK_G88._ Of note, these characteristics of SK_C1 _might be of interest for development of a fibrin-specific version of SK for targeted fibrinolysis^[^^13^^]^. Collectively, these observations might indicate both the contribution of SK2aβ of SK_STAB9023 _and SKαγ of *skcg *(SK_G88_) in PA properties of SK_C1_. Almost the same justification might be considered for SK_C2_ compared to the parental SK, which implys the collaborative contributions of β- and αγ-domains in FG-bound-Plg activation and agrees with proposed negative role of the first 59 residues of SKα in fibrin-dependent mode of the SK action^[^^34^^]^.

Taken together, by molecular exchanging SKβ-domains between groups G and A (SK2a) streptococci and recombinant expression of the two *SK*_Cs_ and two parental SK, we could assess and compare the kinetics and FG-bound-Plg activation of the four SK to gain insights into the role of SKβ and SKαγ in these functional characteristics. To our best of knowledge, this is the first report on domain exchange study between groups G and A streptococci. Our results indicated the minor role of SKβ compared to SKαγ in fibrin-independent amidolytic activity, while the reverse was demonstrated for fibrin-dependent proteolytic activity and FG-bound-Plg activation. The obtained data might be of interest for engineering and development of a fibrin-specific version of SK for targeted fibrinolysis and thrombolytic therapy.
